# Draft Genome Sequence of *Chryseobacterium* sp. JV274 Isolated from Maize Rhizosphere

**DOI:** 10.1128/genomeA.00122-17

**Published:** 2017-04-13

**Authors:** Jordan Vacheron, Audrey Dubost, David Chapulliot, Claire Prigent-Combaret, Daniel Muller

**Affiliations:** UMR, Ecologie Microbienne, CNRS, INRA, VetAgro Sup, UCBL, Université de Lyon, Villeurbanne, France

## Abstract

We report the draft genome sequence of *Chryseobacterium* sp. JV274. This strain was isolated from the rhizosphere of maize during a greenhouse experiment. JV274 harbors genes involved in flexirubin production (*darA* and *darB* genes), bacterial competition (type VI secretion system), and gliding (bacterial motility; type IX secretion system).

## GENOME ANNOUNCEMENT

Bacterial species belonging to the genus *Chryseobacterium* were isolated from various environments such as fishes (1), humans (2), water-cooling systems (3), poultry (4), within production lines of food manufacturing companies (5), etc. In addition, some *Chryseobacterium* strains were isolated from the rhizosphere soils of different plants including potato (6), eggplant (7), wild rye and wild pea (8), cempedak (9), or garden lettuce (10).

*Chryseobacterium* sp. JV274 was isolated from maize rhizosphere cultivated in the natural suppressive soil MS8 in Switzerland in the region of Morens (11). This strain was isolated during an experiment aimed to isolate fluorescent *Pseudomonas* on King’s B media supplemented with chloramphenicol (130 µg mL^−1^), ampicillin (50 µg mL^−1^), and cycloheximid (100 µg mL^−1^) (12). Contrary to currently described *Chryseobacterium* species (1–10), JV274 was able to glide.

DNA extraction was done from an overnight culture using a Nucleospin tissue kit (Macherey Nagel—740952.50, Hoerdt, France). Genomic DNA was sequenced at Mr. DNA (Shallowater, TX, USA) Genome sequencing was performed using MiSeq Illumina technology generating a 2 × 300 bp paired-end library. Sequences were trimmed and used for *de novo* assembling using the NGen DNA assembly software by DNAStar, Inc. The genome annotation was done with the online MicroScope platform (13).

A total of 1,540,972 paired-end reads was obtained giving a coverage depth of 90×. The resulting assemblies generate 19 contigs. The maximum length and *N*_50_ value of the contigs were 1,022,959 bp and 803,304 bp leading to a total length of 5,156,744 bp and an average G+C content of 36.39%. No plasmid was detected for the strain JV274 using the Eckhardt gel technique (14). The JV274 genome harbors 4,819 coding sequences among which 3,124 (64%) are classified in at least one cluster of orthologous group (COG). In addition, 85 tRNAs, 11 rRNAs (three 16S rRNAs, three 23S rRNAs, and five 5S rRNAs) were detected.

This strain possesses two genes (*darA* and *darB*) sharing strong homology with genes involved in the flexirubin production in *Flavobacterium johnsoniae* (up to 60% of identity on 98% of the protein sequences) (15), which may be correlated with its orange pigmentation on solid media (16). In addition, JV274 possesses genes involved in the type VI secretion system (T6SS), in particular, a Vgr-like protein and a PAAR-like effector (CHRYS_v1_170422 and 170423). JV274 also seems to have a T9SS involving, notably, *gldK*, *gldL*, *gldM*, and *gldN* genes (CHRYS_v1_81231 to CHRYS_v1_81234) according to the model described by Abby et al. in 2016 (17), and that might be involved in its gliding motility phenotype. Further studies about T9SS and gene functional analyses will help clarify the gliding mechanisms of *Chryseobacterium* sp. JV274.

### Accession number(s).

This draft genome has been deposited at the European Nucleotide Archive under submission ERA783391, BioProject ID PRJEB18783, sample ERS1496757. Scaffold sequences of the *de novo* assembly have also been deposited at DDBJ/EMBL/GenBank under the accession numbers FTPE01000001 to FTPE01000019.
